# The Effectiveness of Repetitive Transcranial Magnetic Stimulation for Post-stroke Dysphagia: A Systematic Review and Meta-Analysis

**DOI:** 10.3389/fnhum.2022.841781

**Published:** 2022-03-17

**Authors:** Xin Wen, Zicai Liu, Lida Zhong, Yang Peng, Jing Wang, Huiyu Liu, Xiaoqian Gong

**Affiliations:** ^1^Department of Rehabilitation Medicine, Yue Bei People's Hospital, Shaoguan, China; ^2^School of Rehabilitation Medicine Gannan Medical University, Ganzhou, China; ^3^Yue Bei People's Hospital, Shaoguan, China

**Keywords:** stroke, dysphagia, repetitive transcranial magnetic stimulation, meta-analysis, effectiveness

## Abstract

**Background:**

Repetitive transcranial magnetic stimulation (rTMS) applied to the mylohyoid cortical region has positive clinical effects on post-stroke. Therefore, we conducted a meta-analysis to investigate the efficacy of rTMS for patients with post-stroke dysphagia.

**Methods:**

According to PRISMA guidelines, we searched the databases of MEDLINE (PubMed), Cochrane Library, Embase, Web of Science, CNKI, Wangfang. We searched for studies of randomized controlled trials (RCTs) of rTMS to treat dysphagia after stroke and screened by inclusion and exclusion criteria. Features of RCTs were extracted. The heterogeneity of the trials was measured by *I*^2^ statistic.

**Results:**

In total, 11 RCTs with 463 dysphagia patients fulfilled our inclusion criteria. In our analysis, rTMS demonstrated a great beneficial effect for post-stroke dysphagia when combined with traditional swallowing exercises. Moreover, a greatly significant difference (*P* = 0.008) was noted based on stimulation frequency (high frequency vs. low frequency). Additionally, no significant difference (*P* = 0.53) was observed based on stimulation site (affected vs. unaffected hemisphere).

**Conclusions:**

Overall, rTMS can effectively accelerate the improvement of swallowing function in patients with post-stroke swallowing disorders.

## Introduction

Stroke, also known as a cerebrovascular accident, is a common cardiovascular disease with a high prevalence of morbidity, handicap, and fatality worldwide (GBD 2016 Stroke Collaborators, 2019). Deglutition disorders are one of the most widespread post-stroke complications. A previous study (Nepal and Sherpa, 2019) has shown that the prevalence of dysphagia in stroke survivors is up to 37–78%. The prevalence of post-stroke dysphagia reported from different studies also varies, and the prevalence varies by region. Furthermore, an estimated 41% of stroke patients in the United States experience difficulty swallowing (Crary et al., 2013), and nearly 51.14% of stroke inpatients have deglutition disorder in China (Zhang et al., 2021). Post-stroke dysphagia is an impaired swallowing function caused by an imbalance in the coordination of swallowing muscles and neural regulation, which prevents the patients from eating normally (Sandoval-Munoz and Haidar, 2021). Dysphagia is mainly manifested as salivation, coughing from drinking water, and prolonged eating times (Wilkinson et al., 2021). Therefore, patients with swallowing disorders often suffer from complications such as malnutrition, electrolyte disorders, aspiration, aspiration pneumonia, etc (Martino et al., 2005; Shigematsu and Fujishima, 2015). This seriously affects patients' health and quality of life, increases the burden on families and society, and even endangers patients' life safety (Arnold et al., 2016).

The routine treatment measures for swallowing disorders include the following: dietary modification (Reyes-Torres et al., 2019), postural substitution (Terré and Mearin, 2012), physical therapy (Kilinç et al., 2020; Liaw et al., 2020; Lin et al., 2021), acupuncture (Chen and Guo, 2018; Yuan et al., 2019), and sensorimotor stimulation (Simonelli et al., 2019; Wang et al., 2019; Oh et al., 2020). These treatments aim at improving the swallowing function and increasing the speed of eating, but their therapeutic effect is limited to some extent. Although a significant percentage of patients spontaneously regained the ability to swallow in a short period, more than 10% of patients still have residual swallowing problems after routine therapy (Smithard et al., 1997). Therefore, it is particularly important to boost the improvement of swallowing function more effectively in the early phases of stroke and reduce the risk of complications for the recovery of patients.

Hamdy et al. (1998) proposed that the rehabilitation of dysphagia after unilateral stroke was accompanied by an increased excitability of the unaffected hemisphere cortex. In recent years, repetitive transcranial magnetic stimulation (rTMS) has emerged as a treatment modality to enhance swallowing function by modulating cortical excitability (Jefferson et al., 2009; Khedr et al., 2009; Khedr and Abo-Elfetoh, 2010; Wang et al., 2017). It is acknowledged that low-frequency (1 Hz) rTMS has a suppressive effect on the cerebral cortex (Kobayashi et al., 2004; Mansur et al., 2005; Fregni et al., 2006), whereas high-frequency (≥1 Hz) rTMS has an excitatory effect on the cerebral cortex (Pascual-Leone et al., 1998; Peinemann et al., 2004). Lately, several studies (Khedr et al., 2009; Khedr and Abo-Elfetoh, 2010; Park et al., 2017) have demonstrated that rTMS can facilitate the recovery of swallowing function after stroke. However, the effectiveness of rTMS for post-stroke dysphagia is not well-documented by evidence-based medicine. Thus, we here intended to perform a systematic review of the randomized-controlled trials (RCTs) assessing the efficacy of rTMS on post-stroke dysphagia to offer an evidence-based basis for clinical treatment.

## Methods

### Protocol and Registration

Our systematic review was designed and implemented based on the Preferred Reporting Items for Systematic Reviews and Meta-analysis (PRISMA) guideline (Page et al., 2021). The study has been registered with Prospero (CRD42021288484).

### Retrieval Strategy

Associated studies were performed on the databases of Cochrane Library, Embase, MEDLINE (PubMed), CNKI, and Wangfang. We retrieved RCTs of the efficacy of rTMS on post-stroke dysphagia. We did not restrict the language in the search process. The search included the keywords “stroke,” “dysphagia,” “swallowing disorders,” “deglutition disorders,” and “repetitive transcranial magnetic stimulation.” We choose only to included RCTs. In addition, we also manually retrieved some extra trials, which were subject-related and included studies, which were in reviews or meta-analyses. Using Pubmed database as an example, the search strategy was as follows (Table 1).

**Table 1:** The specific search strategy of Pubmed database.

**No**.	**Search items**
1	“Repetitive transcranial magnetic stimulation” [Title/Abstract]
2	“rTMS” [Title/Abstract]
3	1 or 2
4	“Stroke” [Title/Abstract]
5	“Cerebrovascular accident” [Title/Abstract]
6	“Brain vascular accident” [Title/Abstract]
7	4 or 5 or 6
8	“Deglutition disorder” [Title/Abstract]
9	“Deglutition dysfunction” [Title/Abstract]
10	“Dysphagia” [Title/Abstract]
11	“Swallowing disorder” [Title/Abstract]
12	“Swallowing dysfunction” [Title/Abstract]
13	8 or 9 or 10 or 11 or 12
14	randomized controlled trial [publication type]
15	randomized [Title/Abstract]
16	random [Title/Abstract]
17	Controlled [Title/Abstract]
18	14 or 15 or 16 or 17
19	3 and 7 and 13 and 18

### Inclusion and Exclusion Criteria

Only studies with the following criteria were included: (1) the RCT trials with two or more arms, regardless of the sample size of each trial; (2) the peoples were diagnosed with post-stroke dysphagia through clinical examination; and (3) the same interventions were performed in the test and control groups, except that the test groups underwent rTMS. The exclusion criteria were as follows: (1) swallowing disorders are caused by other causes, such as Parkinson's disease, multiple sclerosis, cancer, et al.; (2) duplicated data; (3) inadequate data; and (4) the full text of the studies could not be obtained.

### Study Selection Data Extraction

Firstly, all retrieved studies were imported into the document management system of EndnoteX20, and duplicated studies were deleted. Then, two researchers (WX and LZC) individually read the title and summary based on our inclusion and exclusion criteria. Afterward, we downloaded and read the full text of all studies which have been identified to be relevant and determined the eventually included trials after reading the full text. The inconsistencies during screening were settled through discussions, if necessary, together with another experienced reviewer (LHY).

### Data Extraction and Quality Assessment

Two researchers (LZC and PY) individually extracted data from the included studies. The extraction included author, publication year, sample capacity, age, grouping, interventions, intervention time, measures, efficiency of the rTMS treatment, and so on. Any disagreements during the data collection were resolved by discussions. In addition, we contacted the corresponding author of the study to obtain the original data for the study when the data involved in a study were unclear or difficult to extract.

All included studies were evaluated for risk of bias, which had been done independently by two investigators (LZC and WJ) using the Cochrane risk of bias tool (Revman5.30). Risk bias assessment includes 7 dimensions: random sequence generation, allocation concealment, blinding of participants and personnel, blinding of outcome assessment, incomplete outcome data, selective reporting, and other bias. The discrepancies were resolved through intragroup discussions, if necessary, together with another experienced reviewer (LHY).

### Statistical Analysis

All statistical analyses were conducted with Revman5.30. To evaluate heterogeneity, we utilized an *I*^2^-test. If *I*^2^ < 50%, the fixed-effect model was applied, otherwise, the random-effect model was adapted in the meta-analysis. Because trials did not assess patients using the same outcome indicators, we summarized data using changes pre-and post-intervention across groups. The effect size was expressed by a standardized mean difference (SMD) with a 95% confidence interval (CI).

## Results

### Research Result

**Figure 1:**
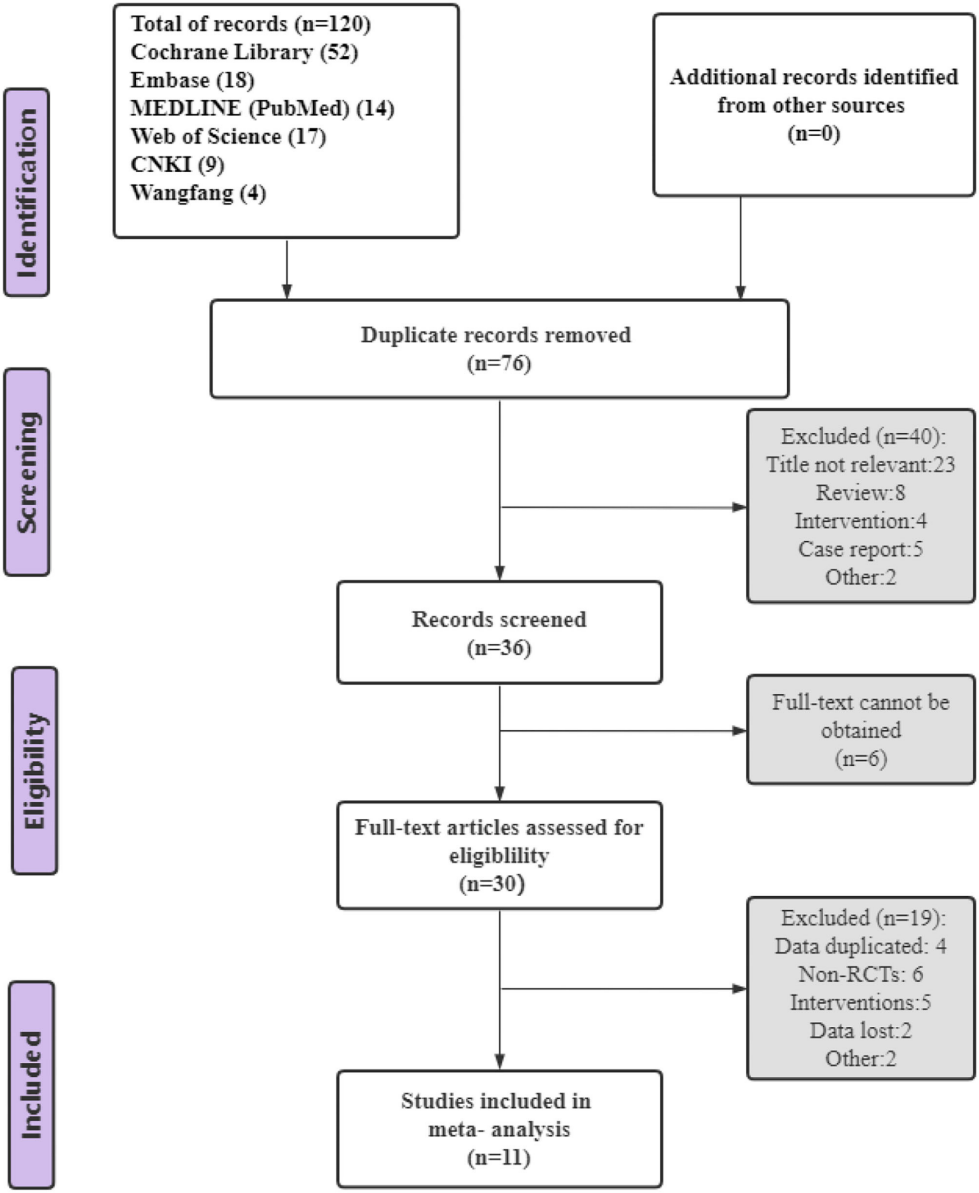
PRISMA flow chart of study selection.

Figure 1 depicts the process of study retrieval and selection. First, a total of 120 studies were identified from all databases, and after removing duplicates, 76 studies remained. Second, 40 studies were excluded after reading the title and summary, 6 were not available in full text, and then 19 were excluded after reading the full text. Eventually, 11 trials were included in our meta-analysis.

### Characteristics of the Trials

Table 2 presents the basic characteristics of the included trials, 11 RCTs with 463 patients, the sample size varied from 18 to 109 and the time after stroke was <6 months. Of these, 293 participants received rTMS and 170 participants only received conventional swallowing treatment. Table 3 shows the main parameters of rTMS, such as frequency, stimulation location, number of pulses, stimulation intensity, and stimulation time. In our meta-analysis, 6 studies (Khedr et al., 2009; Khedr and Abo-Elfetoh, 2010; Kim et al., 2011; Park et al., 2017; Cai et al., 2019; Zhong et al., 2021) used figure-eight coil, 3 studies (Ou-Yang et al., 2019; Zhang et al., 2020; Li et al., 2021) used circular coils, and 2 studies (Lim et al., 2014; Du et al., 2016) did not mention which coils were used.

**Table 2:** The detailed features of the studies.

**Study**	**Patients (F/M)**	**Age (M ± SD)**	**Stroke duration**	**Interventions**	**Total time**	**Outcome**
Zhong et al. (2021)	G1:10/28 G2: 8/28 G3:17/18	G1:64.47 ± 13.95 G2:64.67 ± 10.87 G3:62.34 ± 11.54	<3 months	G1: TST plus rTMS G2: TST plus rTMS G3: TST	2 weeks	PAS scores showed a significantly greater improvement in the G1 and G2 than in the G3.
Li et al. (2021)	G1:5/8 G2:5/7 G3:5/7 G4:4/8	G1:55.7 ± 10.9 G2:57.6 ± 11.4 G3:54.6 ± 8.7 G4: 58.0 ± 10.1	<6 months	G1: TST plus rTMS G2: TST plus rTMS G3: TST plus rTMS G4: TST plus sham stimulation	2 weeks	SSA scores showed a significantly greater improvement in the G1, G2, and G3 than in the G4.
Zhang et al. (2020)	G1:3/11 G2:4/9 G3:4/11	G1:55.21 ± 12.02 G2:56.23 ± 11.89 G3:57.73 ± 15.78	<6 months	G1: TST plus rTMS G2: TST plus rTMS G3: TST plus sham stimulation	2 weeks	PAS scores showed a significantly greater improvement in the G1 and G2 than in the G3 and a greater improvement in the G1 than G2.
Ou-Yang et al. (2019)	G1:7/13 G2:9/11	G1:64.10 ± 12.23 G2:62.50 ± 13.27	2 weeks−3 months	G1: TST plus rTMS G2: TST	2 weeks	PAS scores showed a significantly greater improvement in the G1 than in the G2.
Cai et al. (2019)	G1:5/15 G2:7/13 G3:6/14	G1:63.9 ± 10.9 G2:61.7 ± 9.3 G3:61.1 ± 9.8	1–2 months	G1: TST plus rTMS G2: TST plus rTMS G3: TST plus sham stimulation	2 weeks	SSA scores showed a significantly greater improvement in the G1 and G2 than in the G3, and a greater improvement in the G1 than G2.
Du et al. (2016)	G1:2/13 G2:6/7 G3:6/6	G1:58.2 ± 2.78 G2:57.92 ± 2.47 G3:58.83 ± 3.35	<1 months	G1: TST plus rTMS G2: TST plus rTMS G3: TST plus sham stimulation	5 days	DD scores showed a significantly greater improvement in the G1 and G2 than in the G3.
Lim et al. (2014)	G1:6/9 G2:8/6	G1:62.5 ± 8.2 G2:59.8 ± 11.8	<3 months	G1: TST G2: TST plus rTMS	2 weeks	PAS scores showed a significantly greater improvement in the G2 than in the G1.
Park et al. (2013)	G1:4/5 G2:4/5	G1:73.7 ± 3.8 G2:68.9 ± 9.3	1–4 months	G1: TST plus rTMS G2: TST plus sham stimulation	2 weeks	PAS scores showed a significantly greater improvement in the G1 than in the G2.
Kim et al. (2011)	G1:4/6 G2:5/5 G3:4/6	G1:68.2 ± 12.6 G2:69.8 ± 8.0 G3:66.4 ± 12.3	<3 months	G1: TST plus sham stimulation G2: TST plus rTMS G3: TST plus rTMS	2 weeks	PAS scores showed a significantly greater improvement in the G1 and G2 than in the G3.
Khedr and Abo-Elfetoh (2010)	G1:3/8 G2:3/8	G1:56.14 ± 12.9 G2:59.36 ± 13.6	1–3 months	G1: TST plus rTMS G2: TST plus sham stimulation	5 days	DD scores showed a significantly greater improvement in the G1 than in the G2.
Khedr et al. (2009)	G1:12 G2:14	G1:58.9 ± 11.7 G2:56.2 ± 13.4	<1 months	G1: TST plus rTMS G2: TST plus sham stimulation	5 days	DD scores showed a significantly greater improvement in the G1 than in the G2.

*G, group; F, female; M, male; M ± SD, mean ± standard deviation; TST, traditional swallowing training; rTMS, repetitive transcranial magnetic stimulation; SSA, standardized swallowing assessment; PAS, penetration-aspiration scale; DD, degree of dysphagia*.

**Table 3:** Main parameters of rTMS.

**Study**	**Parameters**				
	**Frequency**	**Stimulation location**	**Intensity**	**Number of pulses a day**	**Stimulation time**
Zhong et al. (2021)	5 HZ	G1: unaffected mylohyoid cortical region G2: affected mylohyoid cortical region	110%RTM	1,800 pulses	20 min a day, 5 days a week for 2 weeks.
Li et al. (2021)	G1:5 HZ G2:3 HZ G3:1 HZ	Unaffected mylohyoid cortical region	120%RTM	G1:2,400 pluses G2:1,440 pulses G3:480 pulses	20 min each time, twice a day, 6 days a week for 2 weeks.
Zhang et al. (2020)	G1:5 HZ G2:1 HZ	Unaffected mylohyoid cortical region	120%RTM	250 pulses	10 min a day, 5 days a week for 2 weeks.
Ou-Yang et al. (2019)	5 HZ	Unaffected mylohyoid cortical region	120%RTM	800 pulses	16 min a day, 7 days a week for 2 weeks.
Cai et al. (2019)	10 HZ	G1: bilateral mylohyoid cortical region G2: affected mylohyoid cortical region	90%RTM	1,000 pulses	20 min a day, 5 days a week for 2 weeks.
Du et al. (2016)	G1:3 HZ G2:1 HZ	G1: affected mylohyoid cortical region G2: unaffected mylohyoid cortical region	90% RTM	1,200 pulses	Once a day for 5 consecutive days.
Lim et al. (2014)	1 HZ	Unaffected pharyngeal motor cortex	100%RTM	1,200 pulses	20 min a day, 5 days a week for 2 weeks.
Park et al. (2013)	5 HZ	Unaffected pharyngeal motor cortex	90% RTM	500 pulses	10 min a day for 2 weeks.
Kim et al. (2011)	G2:5 HZ G3:1 HZ	G2: affected pharyngeal motor cortex G3: unaffected pharyngeal motor cortex	100%RMT	G2:1,000 pulses G3:1,200 pulses	20 min a day, 5 days a week for 2 weeks.
Khedr and Abo-Elfetoh (2010)	3 HZ	Bilateral esophageal motor cortex	130%RTM	600 pulses	20 min every day for five consecutive days.
Khedr et al. (2009)	3 HZ	Affected esophageal motor cortex	120%RTM	300 pulses	10 min every day for five consecutive days.

*rTMS, repetitive transcranial magnetic stimulation; G, group; RTM, resting motor threshold*.

### Quality Assessment Result

**Figure 2:**
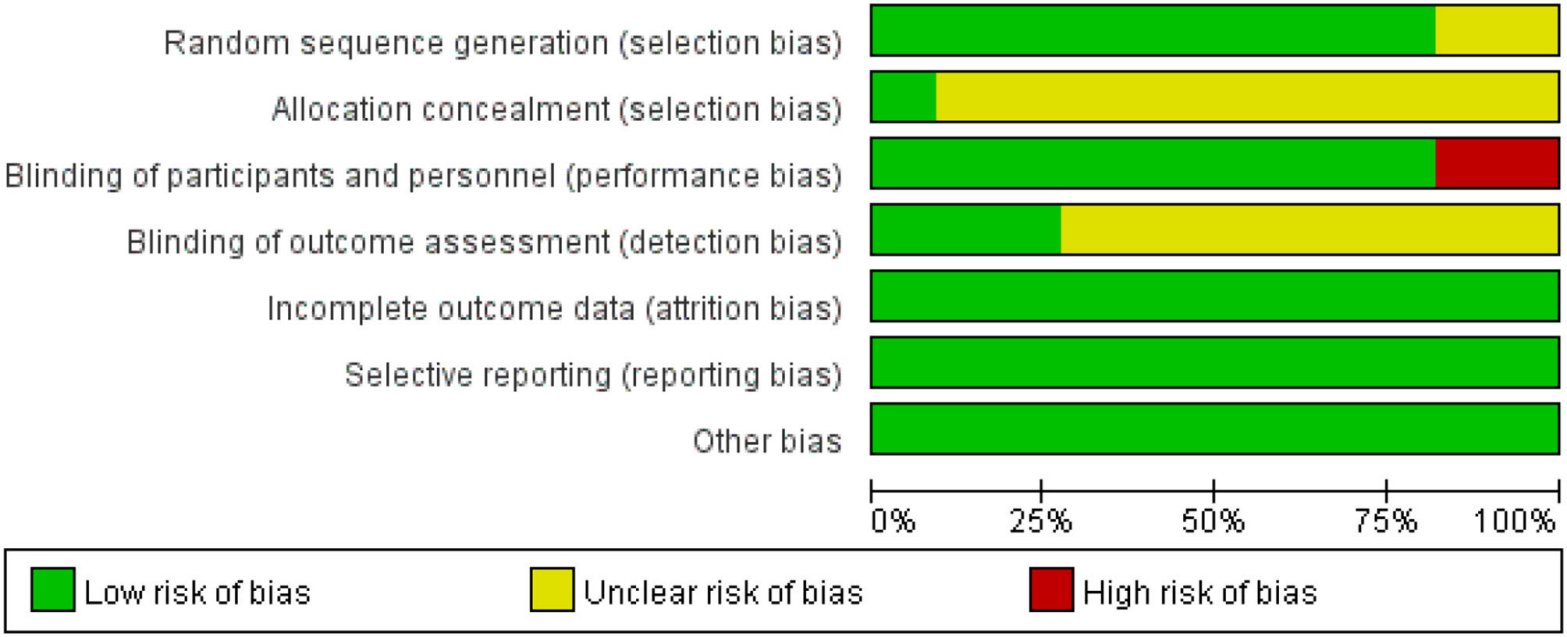
Risk of bias items shown as percentages across the included RCTs.

**Figure 3:**
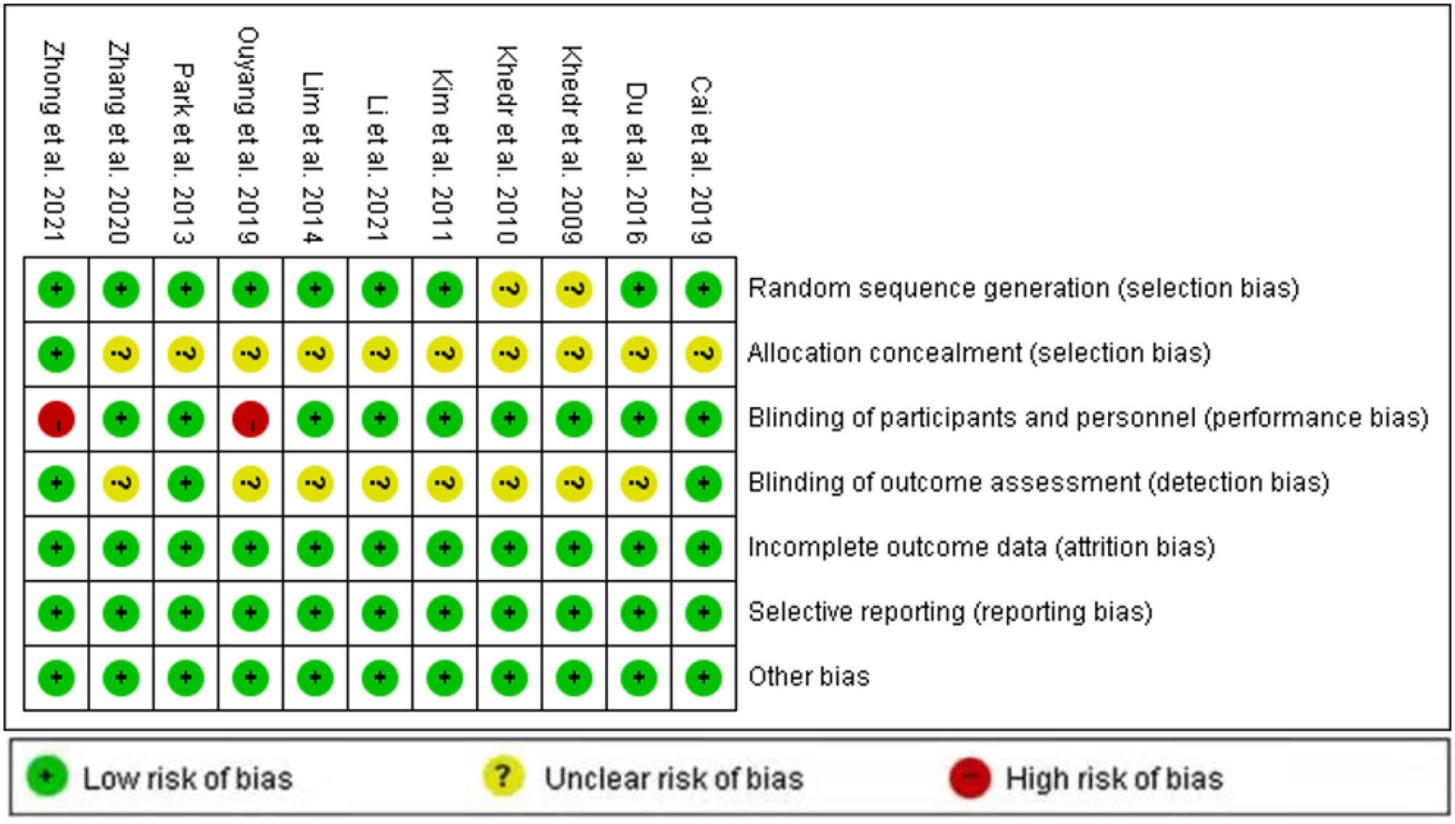
Risk of bias assessment for 11 RCTs.

Figures 2, 3 depict the risk bias assessment according to the Cochrane risk of bias tool (Revman5.30). The risk of selection bias and detection bias was unclear in most studies. However, in general, the risk of bias for the included studies was relatively low.

### Results of Statistical Analysis

**Figure 4:**
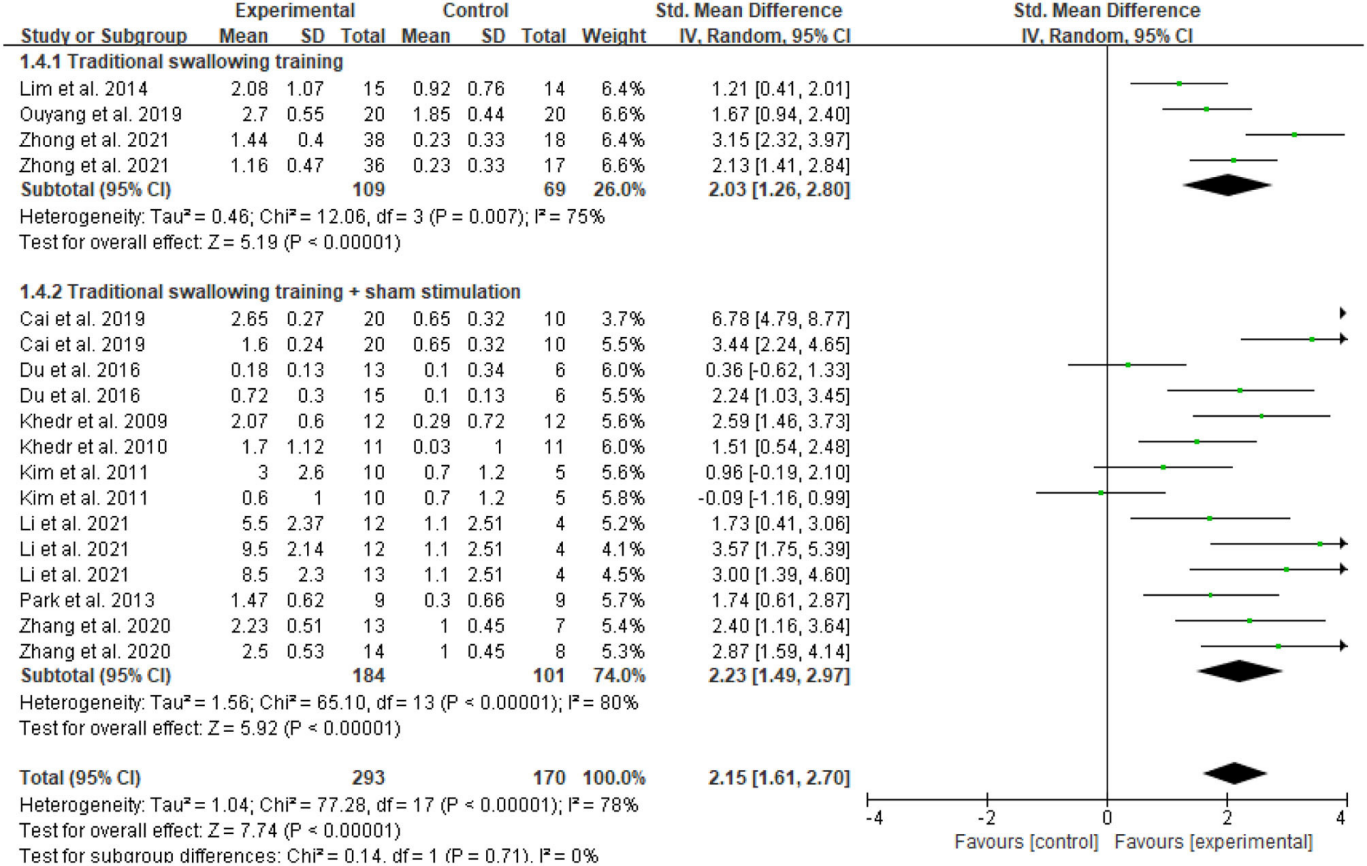
Forest plot of the overall effect analysis of rTMS.

**Figure 5:**
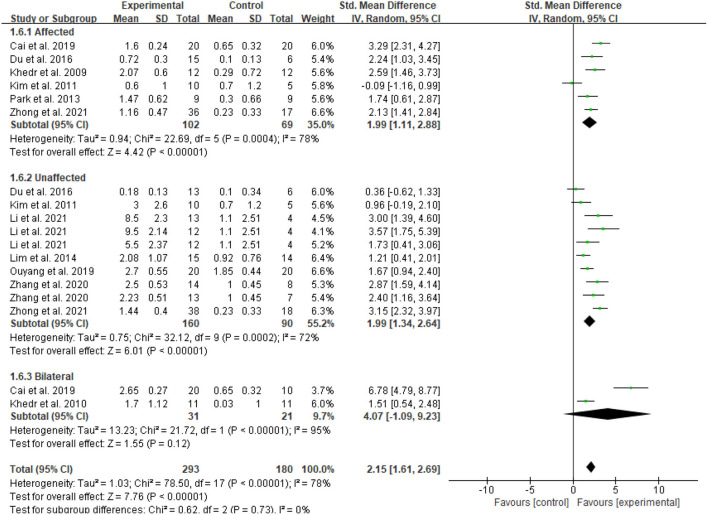
Forest plot for subgroup analysis according to the stimulation site.

**Figure 6:**
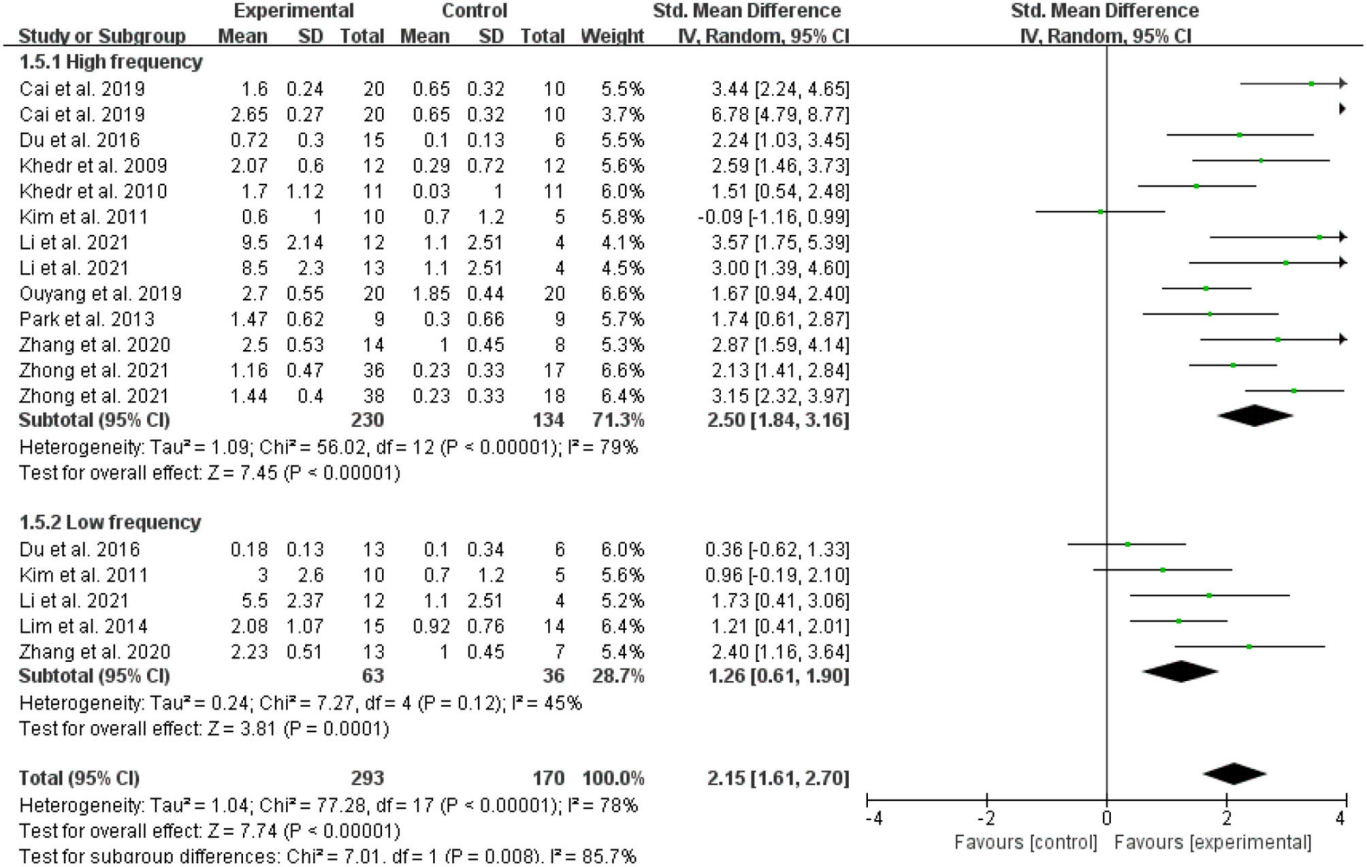
Forest plot for subgroup analysis according to stimulation frequency.

**Figure 7:**
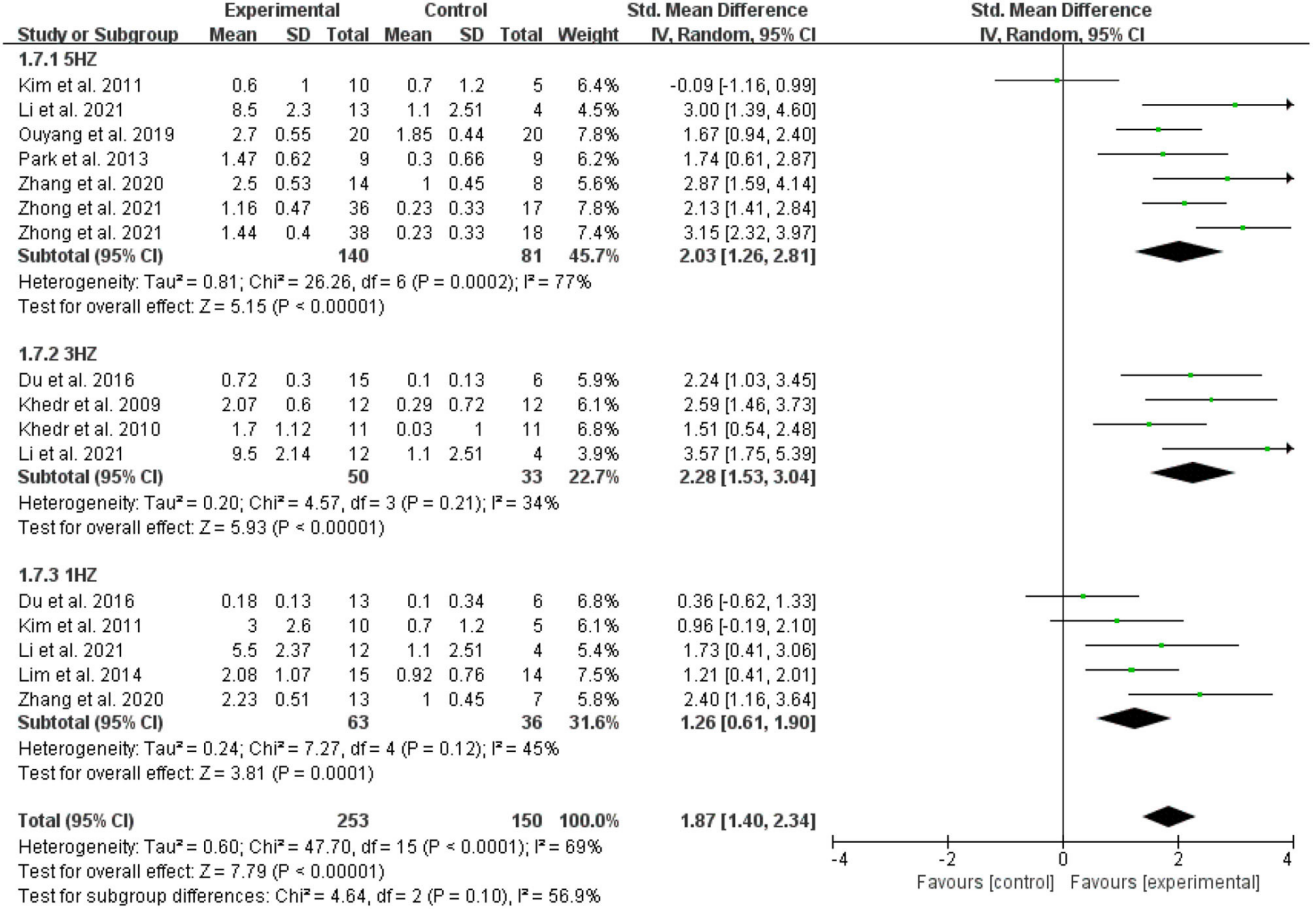
Comparison of the effect size of 5, 3, and 1 Hz.

In the meta-analysis of the 11 trials, statistically significant improvement was found in the experimental group compared with the control group (SMD = 2.15, 95 % CI = 1.61–2.70, *I*^2^ = 78 %; Figure 4). Of the 11 included studies, the control group in three studies performed only standard swallowing training, and the others performed sham stimulation in addition to standard swallowing training. The sham stimulation was the turning of the coils 180° instead of using a sham coil. The active stimulation had better therapeutic effects compared to either the conventional swallowing training or the sham stimulation, and a statistically significant difference was found between traditional swallowing training and placebo treatment. The stimulation of the affected or unaffected hemisphere and bilateral stimulation had better therapeutic effects compared to either the conventional swallowing training or the sham stimulation (SMD = 2.15, 95% CI = 1.61–2.69, *I*^2^ = 78%; Figure 5). However, no statistically significant difference was found between stimulating the affected hemisphere vs. the unaffected hemisphere vs. bilateral stimulation (*I*^2^ = 0%, *p* = 0.73; Figure 5). The subgroup analysis based on frequency revealed greatly significant improvement (SMD = 2.15, 95% CI = 1.61–2.70, *I*^2^ = 78%; Figure 6), and a statistically significant difference was found between the high-frequency group and low-frequency group (*I*^2^ = 85.7 %, *p* = 0.008; Figure 6). This analysis found that the effect size of 3 HZ was the largest (SMD = 2.28, 95% CI = 1.53–3.04, *I*^2^ = 34%; Figure 7) and the effect size of 1 HZ was the smallest (SMD = 1.26, 95% CI = 0.61–1.90, *I*^2^ = 45%; Figure 7), but there was no significant difference between the three groups of 1, 3, and 5 Hz (*I*^2^ = 56.9 %, *p* = 0.1; Figure 7).

## Discussion

The results of this quantitative analysis showed that the overall effects of rTMS on the recovery of swallowing function were robust. This result was consistent with that of previous studies (Yang et al., 2015; Pisegna et al., 2016; Liao et al., 2017), which have reported that rTMS can efficiently contribute to the rehabilitation of swallowing function. Several studies have shown that both high-frequency and low-frequency stimulation can contribute to the improvement of swallowing function after stroke (Khedr et al., 2009; Verin and Leroi, 2009; Khedr and Abo-Elfetoh, 2010; Cheng et al., 2017b; Park et al., 2017; Ünlüer et al., 2019).

In addition, the result of subgroup analysis demonstrated greater effect sizes for stimulation of affected, unaffected, or bilateral hemisphere compared to standard swallowing training or placebo treatment. However, there were no significant differences between stimulating the affected hemisphere vs. the unaffected hemisphere vs. bilateral stimulation. A study by Zhong et al. (2021) proposed that 5 Hz-rTMS on either the unaffected or affected hemisphere improved post-stroke dysphagia, but there was no significant difference between the two. Cai et al. (2019) reported that bilateral stimulation is more efficient than unilateral stimulation in enhancing swallowing function after stroke. A case report (Momosaki et al., 2014) suggested bilateral stimulation could effectively facilitate the rehabilitation of post-stroke dysphagia. Similar results were reported by Park et al. (2013). However, a meta-analysis including the present RCTs gave not sufficient support that bilateral stimulation is more effective than unilateral. It has been shown that the pharyngeal cortical regions are presented in the bilateral cerebral hemispheres (Hamdy et al., 1996), it is now generally accepted that the swallowing functional areas in the cerebral cortex are found to have significant interhemispheric asymmetry (Wilmskoetter et al., 2018). Therefore, stimulation in either the healthy or the affected hemisphere improves swallowing function after stroke. Swallowing disorders occur when the “dominant” hemisphere is damaged and the unaffected non-dominant center is unable to compensate. Most patients with dysphagia may have damage to the dominant hemisphere of swallowing, and fewer relevant connections between the swallowing cortical networks of the damaged hemisphere may remain, so the recovery of swallowing function may be dependent on the unaffected side (Ou-Yang et al., 2019). Similarly, some researchers (Liao et al., 2017) also believed that stimulation on the unaffected hemisphere was superior to that on the affected hemisphere, but our study did not find this result. Khedr and Abo-Elfetoh (2010) and Cai et al. (2019) also thought that stimulation on the bilateral hemisphere was effective. In addition, a study on healthy volunteers by Sasegbon et al. (2021). found an inhibitory effect of high-frequency rTMS in the cerebellar vermis on pharyngeal motor cortical activity and swallowing behavior. Similarly, Zhong et al. (2021). showed that high-frequency rTMS applied to the cerebellum was effective in improving swallowing function in stroke patients. Furthermore, Lin et al. (2018) placed the coil of rTMS on the left postauricular mastoid in patients with brainstem injury to stimulate their vagus nerve, and found that the swallowing function of patients in the real stimulation group was significantly improved compared with the sham stimulation group. In conclusion, rTMS applied to the mylohyoid cortical region, pharyngeal motor cortex, esophageal motor cortex, cerebellum, and the left postauricular mastoid are all helpful in improving swallowing function. However, for the available studies, the cortical area of the mylohyoid muscle has been most frequently selected and has been noted to be effective. Gallas et al. (2007) thought that swallow recovery is associated with the cortical representation and mylohyoid pathways of swallowing muscle. Hamdy et al. (1996) found that mylohyoid EMG response amplitudes were larger than those of the pharyngeal response and esophageal response. We speculate that the amplitudes of the EMG response in the cortical region of the mylohyoid muscle are more obvious than others, and stimulation on the mylohyoid cortical region is also effective, so the mylohyoid cortical region is the most frequently stimulated site. Currently, there are many stimulation sites for dysphagia, and no studies have been conducted to compare its therapeutic efficacy, and the selection of the optimal stimulation site and its accurate localization are crucial to improve treatment for dysphagia. Therefore, future studies are required to investigate the effect of different stimulation sites on the therapeutic efficacy in order to find the optimal stimulation site and provide a basis for clinical treatment.

Furthermore, subgroup analyses based on frequency also revealed that high-frequency stimulation has a greater effect size than low-frequency stimulation. This result was similar to the previous review by Liao et al. (2017). Kim et al. (2011) thought that high-frequency rTMS on the affected hemisphere did not produce changes in swallowing function, while Khedr and Abo-Elfetoh (2010) and Cai et al. (2019) believed that high-frequency stimulation on the affected hemisphere had a significant benefit for post-stroke dysphagia. Ünlüer et al. (2019) found that 1 Hz rTMS promoted the recovery of swallowing function in the subacute period. It was usually believed that high frequency was typically used for the affected side increases cortical excitability, while the low frequency was typically used for the unaffected side decreases its excitability and equalizes cortical excitability in bilateral hemispheres, thus improving swallowing function. Hamdy et al. (2000) concluded that the recovery of post-stroke dysphagia depends on the functional compensation of the unaffected side, thus high-frequency stimulation on the unaffected hemisphere also could improve the swallowing function of patients. Unfortunately, all stroke patients involved in the included studies were in the acute or subacute period, and there were not enough RCTs for the chronic period. As a result, this meta-analysis can only analyze the effect of rTMS on the recovery of swallowing function in patients with acute or subacute stroke, but not on the recovery of swallowing function in patients with chronic stroke. A pilot study by Verin and Leroi (2009) suggested that 1 Hz rTMS improved swallowing function in stroke patients with a disease duration of more than 6 months, and since there was no sham and control group in this study, it could only show that low-frequency rTMS was beneficial for swallowing function recovery, but could not confirm that rTMS was more beneficial than the conventional swallowing training. Thus, they couldn't state whether rTMS was effective in improving swallowing function in patients with chronic post-stroke dysphagia. However, Cheng et al. (2017a) revealed that 5 Hz rTMS did not improve swallowing function compared to placebo treatment in chronic (>12 months) dysphagia. The reason why rTMS did not promote the recovery of swallowing function in patients in the chronic period may be that most selected participants with chronic post-stroke dysphagia have mild to moderate dysphagia, and there was little room for improvement in swallowing function, or it may be because the patients had extensive damage to the pharyngeal cortical areas of the bilateral cerebral hemispheres, and the effect was not significant regardless of the treatment measures. No studies have proven that there is an optimal intervention period during which rTMS is most effective, while beyond the optimal intervention period, rTMS is not effective. In addition, as rTMS is being used more frequently to treat post-stroke dysphagia, studies have begun to investigate whether different stimulation frequencies affect the treatment effect. Stimulation frequencies of 1, 3, and 5 Hz were used in the study. Considering the here included studies, 5 Hz is the most frequent and effective stimulation frequency. It was found that different stimulation frequencies would have different therapeutic effects as well. Therefore, to investigate the optimal stimulation frequency for rTMS, a subgroup analysis was performed. There was no significant difference in the effectiveness of 1, 3, and 5 Hz. However, a study by Li et al. (2021) also observed that the improvement of swallowing function after stroke was more obvious in 5 Hz rTMS compared to 3 Hz rTMS. Therefore, we could not determine which one is more effective, 5 Hz rTMS or 3Hz rTMS, and more studies can be done to directly compare the efficacy between them.

Fitzgerald et al. (2002) used 1 Hz rTMS of different stimulation intensities treatment on healthy volunteers, and found that the stimulation intensity of 115% resting motor threshold (RMT) was more effective than 85% RMT in inhibiting cortical excitability. A similar study was done by Mistry et al. (2007) they also used 1 Hz rTMS of different stimulation intensity treatments on patients with dysphagia and found that the stimulation intensity of 120% RMT was more effective than 80% RMT in improving the swallowing function of patients. Both studies were given low-frequency rTMS and compared the effects of different stimulation intensities on cortical excitability. There is no study regarding the effect of different stimulation intensities on cortical excitability with high-frequency rTMS, nor regarding the effect of the same stimulation intensity on the excitability with different stimulation locations. A study (Modugno et al., 2001) demonstrated that the greater the number of stimulus pulses, the wider the excitation of the cerebral cortex. Gilio et al. (2007) stimulated healthy subjects with different numbers of pulses of 5 Hz rTMS, and found that when the number of pulses was low, even when high-frequency stimulation was given, it did not excite the cerebral cortex, but rather inhibited it. It is generally believed that more pulses will have a more long-lasting benefit compared to fewer pulses, regardless of other stimulation factors. At present, there is no clinical study comparing rTMS with the different number of pulses for post-stroke dysphagia, nor is there a study comparing the effect of different stimulation times on the therapeutic effect, even though many articles do not explicitly state the stimulation time. A study by Khedr and Abo-Elfetoh (2010) found that rTMS plays an important role in the recovery of dysphagia in patients with lateral medullary syndrome and brainstem infarction. Otherwise, most studies have grouped patients with different stroke types for analysis, and no studies have investigated the effects of rTMS on patients with different stroke types. Therefore, future studies are required to investigate the effects of the different number of pulses and different stimulation times on the therapeutic efficacy, and to explore the effects of rTMS on patients with different stroke types.

Although we provided general information of currently available evidence for the application of rTMS in post-stroke dysphagia, our meta-analysis also had some limitations. First, TMS technology has some inherent limitations, for example, the localization of the stimulation site may not be very accurate, which has an impact on the treatment effect. The risk of seizures induced by TMS is about <0.03% (Rossi et al., 2021), and high-frequency TMS in the area of an acute or subacute stroke would increase the seizure risk. In addition, other limitations inherent to TMS technology include difficult delivery of a meaningful sham condition and high placebo response, inability to access non-cortical/deeper targets, lack of access to TMS at institutions, lack of portability, etc. Second, compared with the previous meta-analysis (Liao et al., 2017), more RCTs were included in this study, but the number of studies and patients included was relatively small. Third, different swallowing scales were used to assess the improvement of swallowing function, which may cause variations in the results. Finally, the heterogeneity across the included studies was relatively obvious, which may cause variations in the results. The reason for this high heterogeneity may be due to the different types of stroke patients, the different periods of stroke patients, and the different stimulation options of rTMS between studies.

## Conclusion

Overall, rTMS can effectively promote the recovery of swallowing function in patients with post-stroke dysphagia. In our analysis, rTMS demonstrated a great beneficial effect for post-stroke dysphagia when combined with traditional swallowing exercises. Moreover, our findings suggested that high-frequency rTMS is more effective than low frequency. However, considering the high heterogeneity in this meta-analysis, we couldn't yet clear whether low-frequency stimulation is necessarily superior to high-frequency stimulation, and more RCTs need to be included for further analysis in the future. Additionally, no significant difference based on stimulation site (affected, unaffected and bilateral stimulation) was observed. In the future, more RCTs are needed to investigate the effect of different stimulation sites, different stimulation frequencies, different stimulation intensities, the different number of pulses, and different stimulation times on the therapeutic efficacy to find the optimal stimulation parameters and develop a personalized treatment plan for patients.

## Data Availability Statement

The datasets presented in this study can be found in online repositories. The names of the repository/repositories and accession number(s) can be found in the article/supplementary material.

## Author Contributions

HL: conceptualization, writing-reviewing and editing, data extraction, and assessing the risk of bias. XW: writing-original draft, study selection, research retrieval, and statistical analysis. ZL: study selection, data extraction, and writing-reviewing. LZ: article revision and writing-reviewing. YP: data extraction. JW: assessing the quality of studies. XG: article revision and grammar revision. All authors contributed to the article and approved the submitted version.

## Conflict of Interest

The authors declare that the research was conducted in the absence of any commercial or financial relationships that could be construed as a potential conflict of interest.

## Publisher's Note

All claims expressed in this article are solely those of the authors and do not necessarily represent those of their affiliated organizations, or those of the publisher, the editors and the reviewers. Any product that may be evaluated in this article, or claim that may be made by its manufacturer, is not guaranteed or endorsed by the publisher.
